# Long-Term Exposure to Ambient PM_2.5_, Sunlight, and Obesity: A Nationwide Study in China

**DOI:** 10.3389/fendo.2021.790294

**Published:** 2022-01-07

**Authors:** Rui Chen, Chao Yang, Pengfei Li, Jinwei Wang, Ze Liang, Wanzhou Wang, Yueyao Wang, Chenyu Liang, Ruogu Meng, Huai-yu Wang, Suyuan Peng, Xiaoyu Sun, Zaiming Su, Guilan Kong, Yang Wang, Luxia Zhang

**Affiliations:** ^1^ Renal Division, Department of Medicine, Peking University First Hospital, Peking University Institute of Nephrology, Beijing, China; ^2^ Research Units of Diagnosis and Treatment of Immune-mediated Kidney Diseases, Chinese Academy of Medical Sciences, Beijing, China; ^3^ Advanced Institute of Information Technology, Peking University, Hangzhou, China; ^4^ Key Laboratory for Earth Surface Processes of the Ministry of Education, College of Urban and Environmental Sciences, Peking University, Beijing, China; ^5^ School of Public Health, Peking University, Beijing, China; ^6^ National Institute of Health Data Science at Peking University, Beijing, China; ^7^ National Climate Center, China Meteorological Administration, Beijing, China

**Keywords:** obesity, abdominal obesity, PM_2.5_ concentration, sunlight, air pollution

## Abstract

**Background:**

Accumulated researches revealed that both fine particulate matter (PM_2.5_) and sunlight exposure may be a risk factor for obesity, while researches regarding the potential effect modification by sunlight exposure on the relationship between PM_2.5_ and obesity are limited. We aim to investigate whether the effect of PM_2.5_ on obesity is affected by sunlight exposure among the general population in China.

**Methods:**

A sample of 47,204 adults in China was included. Obesity and abdominal obesity were assessed based on body mass index, waist circumference and waist-to-hip ratio, respectively. The five-year exposure to PM_2.5_ and sunlight were accessed using the multi-source satellite products and a geochemical transport model. The relationship between PM_2.5_, sunshine duration, and the obesity or abdominal obesity risk was evaluated using the general additive model.

**Results:**

The proportion of obesity and abdominal obesity was 12.6% and 26.8%, respectively. Levels of long-term PM_2.5_ ranged from 13.2 to 72.1 μg/m^3^ with the mean of 46.6 μg/m^3^. Each 10 μg/m^3^ rise in PM_2.5_ was related to a higher obesity risk [OR 1.12 (95% CI 1.09-1.14)] and abdominal obesity [OR 1.10 (95% CI 1.07-1.13)]. The association between PM_2.5_ and obesity varied according to sunshine duration, with the highest ORs of 1.56 (95% CI 1.28-1.91) for obesity and 1.66 (95% CI 1.34-2.07) for abdominal obesity in the bottom quartile of sunlight exposure (3.21-5.34 hours/day).

**Conclusion:**

Long-term PM_2.5_ effect on obesity risk among the general Chinese population are influenced by sunlight exposure. More attention might be paid to reduce the adverse impacts of exposure to air pollution under short sunshine duration conditions.

## Introduction

Obesity represents a severe public health challenge globally. The prevalence of obesity came to a high level recently, exceeding 13% globally, and contributed to a decline in both quality of life and life expectancy (1–3). The Nutrition and Chronic Disease Status of Chinese Residents (2020) estimated that 16.4% of the Chinese adult residents were obese, and obesity prevalence was increasing (4). Obesity has been attributed to behavioral, genetic, socioeconomic, and environmental factors. Furthermore, air pollution has been considered as one of the main environmental causes affecting obesity (5).

Ambient fine particulate matter (PM_2.5_) has emerged as a major air pollution globally. A recent cross-sectional research of 2660 children suggested that PM_2.5_ was positively related to a high obesity risk (6). Meanwhile, meteorological factors, such as sunlight, are also regarded as novel potential environmental risk factors for obesity. Previous studies on sunlight exposure have shown that sunlight exposure decreased the risk of obesity (7). Recent *in vitro* and animal experiments have indicated that PM_2.5_ and limited sunlight exposure have several physiological effects in common, including systematic inflammation, insulin resistance, and stimulation of the differentiation of pre-adipocytes *via* reduction of serum vitamin D (8). These effects are all potentially linked with the pathogenesis of obesity. Therefore, it is far more likely that sunlight could modified the contribution of PM_2.5_ to obesity. Nevertheless, evidence for the potential impact of PM_2.5_ on obesity under different sunlight conditions is limited. Moreover, for countries with relatively high levels of PM_2.5_ like China, it is necessary to assess the sunlight effect on the relationship of PM_2.5_ with obesity.

Therefore, the research aimed to examine the relationship of PM_2.5_ and sunlight exposure with obesity risk among the general population in China using a national representative sample.

## Material and Methods

### Study Population

A sample of the general Chinese residents aged ≥18 years was obtained from September 2009 to September 2010, using a multistage, stratified, probability-proportional-to-size sampling method. We obtained participants from 13 provinces (Beijing, Sichuan, Inner Mongolia Autonomous Region, Jiangsu, Xinjiang Uyghur Autonomous Region, Ningxia Hui Autonomous Region, Zhejiang, Guangxi Zhuang Autonomous Region, Guangdong, Shanghai, Hubei, Hunan, and Shandong) in China. Information on participants’ sociodemographic status, lifestyle, and health history was obtained. Each questionnaire and on-site examination including anthropometric measurement was finished at community medical centers or hospitals by medical students, trained primary care physicians, and nurse practitioners. Detailed data collection and measuring methods have been mentioned previously (9). The study population for this survey included 47,204 participants with completed questionnaire and health examinations, and recruitment was conducted when participants’ addresses could be well-followed. The study was conducted in accordance with the Declaration of Helsinki (as revised in 2013). Each subject provided informed written consent before data collection. The ethics committee at Peking University First Hospital approved the study (Approval number: [2007]056).

### Outcomes

Anthropometric measurements (weight, height, waist and hip circumference) were performed by the staff members using standardized procedures. The body mass index (BMI) was calculated by dividing weight (kg) by height squared (m^2^). BMI was categorized into non-obesity (BMI <28 kg/m^2^), and obesity (BMI ≥28 kg/m^2^). Waist circumference (WC) and waist-to-hip ratio (WHR) were categorized into non-abdominal obesity (WC<90 cm/WHR<0.9 for men; WC<80 cm/WHR<0.8 for women), and abdominal obesity (WC≥90 cm/WHR≥0.9 for men; WC≥80 cm/WHR≥0.8 for women) (10).

### Exposure

Air pollution monitoring data were collected from satellite remote sensing (SRS) based on aerosol optical depth data obtained from multi-source satellite products (multi-angle imaging spectroradiometer, moderate resolution imaging spectroradiometer) and a geochemical transport model (11–13). PM_2.5_ levels were assessed from PM_2.5_ concentration map products obtained by SRS at a spatial resolution of 1 km.

The meteorologic data such as sunshine duration was derived from surface meteorological observations in China’s meteorological stations obtained from the Surface Meteorological Observation Practice and the Nationwide Surface Climate Data Statistic Method (14–16). Sunshine duration was used to describe the period in a day when the intensity of the direct insolation reaches an average of 120 watts/meters^2^.

Annual individual PM_2.5_ exposure values and sunshine duration before the survey date were assigned to participants based on each participant’s residential address at the street level, which was geocoded into latitude and longitude. The five-year mean PM_2.5_ concentration and sunshine duration prior to the survey date were calculated as the primary exposure variables in our analysis. The details of the calculation of PM_2.5_ indicator and sunshine duration were presented previously (13, 17, 18).

### Assessment of Other Covariates

Information on participants’ sociodemographic features (age, sex, household income, and educational background), life behaviors (current smoking, intakes of alcohol, exercise duration, fruit and vegetable diet, and daily protein intake), urban or rural residence, the annual exposure level of nitrogen dioxide was collected.

### Statistical Analysis

Characteristics for people with obesity or abdominal obesity were reported as percentages or mean (standard deviation, SD). A comparison between those with and without obesity and abdominal obesity was conducted using t test or Wilcoxon rank-sum test (continuous variables), and Chi-squared test (categorical variables).

The general additive model (GAM) was applied to examine the obesity risk and abdominal obesity associated with an elevation of 10 μg/m^3^ in the level of long-term exposure to PM_2.5_. We included potential confounders that have been previously related to obesity. These covariates included age (continuous), sex (male/female), household income (low-income, middle-income, or high-income), educational background (≥high school versus <high school), rural (yes/no), current smoking (yes/no), and intakes of alcohol (never, five times per week to once per month, or almost once a day), the annual exposure level of nitrogen dioxide (continuous), and sunshine duration (continuous).

The interaction between PM_2.5_ and sunshine duration for the risk of obesity and abdominal obesity was tested. This was accomplished by including their multiplicative interaction term in the GAM, given the *a priori* hypothesized relationship between these two factors. Then the stratified analyses were performed if the significant interaction was identified. The relationship between PM_2.5_ and obesity or abdominal obesity risk across different sunshine duration strata (as quartiles) was assessed.

We performed sensitivity analyses to assess whether the outcomes were robust. We used one-year average PM_2.5_ instead of five-year PM_2.5_ and adjusted for varied covariates in the analysis, including exercise duration, fruit and vegetable diet, and daily protein intake as potential confounders. Logistics models were applied to assess the association instead of GAM. We also performed stratified analyses by south/north and longitude (UTC+7, UTC+8) to control the influence of PM_2.5_ composition on obesity.

The *P*<0.05 (two-sided) was statistically significant. All analyses were conducted by SAS 9.4.

## Results

Participants Characteristics are presented in **Table 1**. Among 47,204 participants, 5,940 (12.6%) and 12,629 (26.8%) were obese and abdominal obese, respectively. Over half of the subjects were women (55.0%), and the mean age was 49.6 ± 15.2 years. Compared with non-obesity participants, those with obesity were more likely to be older, have less education, smoke, drink alcohol frequently.

**Table 1:** Characteristics of the study population stratified by obesity or abdominal obesity.

	Total		Obesity				*P*-value^a^	Abdominal Obesity				*P*-value^b^
			Non-obesity		Obesity			Non-abdominal obesity		Abdominal obesity		
	N	Mean (SD)/Median (IQR)/percentage	N	Mean (SD)/Median (IQR)/percentage	N	Mean (SD)/Median (IQR)/percentage		N	Mean (SD)/Median (IQR)/percentage	N	Mean (SD)/Median (IQR)/percentage	
**Age (years)**	47204	49.60 (15.21)	41264	49.17 (15.36)	5940	52.54 (13.84)	<0.001	34575	48.12 (15.28)	12629	53.64 (14.28)	<0.001
**Sex, %**							0.881					0.111
Male	20148	42.7	17606	42.7	2542	42.8		14683	42.5	5465	43.3	
Female	27056	57.3	23658	57.3	3398	57.2		19892	57.5	7164	56.7	
**Education**							<0.001					<0.001
≥high school	20950	44.4	18531	44.9	2419	40.7		16021	46.3	4929	39.0	
<high school	26254	55.6	22733	55.1	3521	59.3		18554	53.7	7700	61.0	
**Family income**							<0.001					<0.001
Low-income	13458	28.5	11772	28.5	1686	28.4		9735	28.2	3723	29.5	
Middle-income	29410	62.3	25621	62.1	3789	63.8		21534	62.3	7876	62.4	
High-income	4336	9.2	3871	9.4	465	7.8		3306	9.5	1030	8.1	
**Rural**	21859	46.3	19599	47.5	2260	44.8	<0.001	16099	46.6	5760	45.6	0.115
**Current smoker**	11094	23.5	9629	23.3	1465	24.7	0.039	7941	23.0	3156	25.0	<0.001
**Drinking**							<0.001					<0.001
Never	35706	75.6	31420	76.1	4286	72.1		26485	76.6	9221	73.0	
five times per week to once per month	6774	14.4	5771	14.0	1003	16.9		4834	14.0	1940	15.4	
Almost once a day	4724	10.0	4073	9.9	651	11.0		3256	9.4	1468	11.6	
**PM_2.5_ (μg/m^3^)**	47204	46.62 (15.51)					<0.001					<0.001
Q1 (13.20-40.82)	11822		10383	87.9	1439	12.1		8600	72.7	3222	27.3	
Q2 (40.82-47.84)	11523		10401	90.3	1122	9.7		9067	78.7	2456	21.3	
Q3 (47.84-56.49)	11994		10548	87.9	1446	12.1		8716	72.7	3278	27.3	
Q4 (56.49-72.13)	11865		9932	83.7	1933	16.3		8192	69.0	3673	31.0	
**Sunshine duration (h/d)**	47204	6.93 (1.61)					<0.001					<0.001
Q1 (3.21-5.34)	12127		11237	92.7	890	7.3		10313	85.0	1814	15.0	
Q2 (5.34-7.18)	12257		11038	90.1	1219	9.9		9581	78.2	2676	21.8	
Q3 (7.18-8.37)	10525		8300	78.9	2225	21.1		6286	59.7	4239	40.3	
Q4 (8.37-9.30)	12295		10689	86.9	1606	13.1		8395	68.3	3900	31.7	

Data are presented as n (percentage) or mean (SD) or median (IQR).

PM_2.5_, fine particulate matter; SD, standard deviation; IQR, interquartile; Q, quartile.

^a^Overweight group and obesity group was compared with normal weight group, respectively.

^b^Abdominal obesity group was compared with non-abdominal obesity group.

Wilcoxon rank-sum test was used for numerical variable and Chi-square test for categorical variable.

Estimated PM_2.5_ levels ranged from 13.20 μg/m^3^ to 72.13 μg/m^3^ for five-year exposure, and the overall mean ambient PM_2.5_ concentration in the study population reached 46.62 μg/m^3^ (SD of 15.51 μg/m^3^). The overall mean five-year sunshine duration was 6.93 hours (SD of 1.61 hours) per day. The PM_2.5_ and sunshine duration levels by each province were diverse (**Table S1**).

The relationship of PM_2.5_ exposure with obesity risk and abdominal obesity risk was significant after adjusting for potential confounding factors (**Table 2**). A five-year average PM_2.5_ level increase of 10 μg/m^3^ was positively related to obesity risk [OR 1.12 (95% CI, 1.09-1.14)]. A positively significant association was also observed for abdominal obesity [OR 1.10 (95% CI, 1.07-1.13)].

**Table 2:** Estimated effects of 5-year mean PM_2.5_ (10μg/m^3^) on the risk of obesity and abdominal obesity in China.

	Obesity		Abdominal obesity		
	OR (95% CI)	*P*-value	OR (95% CI)		*P*-value
**Overall**					
Crude OR	1.07 (1.05,1.10)	<0.001	1.03 (1.02,1.04)		<0.001
Model 1	1.06 (1.04,1.09)	<0.001	1.01 (0.99,1.03)		0.332
Model 2	1.03 (1.01,1.05)	0.003	1.01 (0.99,1.03)		0.199
Model 3	1.12 (1.09,1.14)	<0.001	1.10 (1.07,1.13)		<0.001
**Subgroup**					
**Sunshine duration, h/d**					
Q1(3.21-5.34)	1.56 (1.28,1.91)	<0.001	1.66 (1.34,2.07)		<0.001
Q2(5.34-7.18)	1.34 (1.22,1.47)	<0.001	1.42 (1.27,1.58)		<0.001
Q3(7.18-8.37)	1.02 (0.97,1.08)	0.382	0.99 (0.94,1.05)		0.774
Q4(8.37-9.30)	1.04 (1.00,1.08)	0.027	1.04 (1.00,1.08)		0.053

Model 1: Age, sex, and NO_2_.

Model 2: Age, sex, educational background, smoker, intake of alcohol, household income, rural, and NO_2_.

Model 3: Age, sex, educational background, smoker, intake of alcohol, household income, rural, NO_2_, and sunlight hours.

We observed an inverse J-shaped relationship of PM_2.5_ level with risk of obesity/abdominal obesity as the sunshine duration increased (**Table 2**; *P* value for interaction <0.001), with the lowest risk at 7.18-8.37 h/d. The highest effect estimates of PM_2.5_ for obesity (OR 1.56; 95%CI, 1.28-1.91) and abdominal obesity (OR 1.66; 95% CI, 1.34-2.07) were observed in the bottom quartile of sunlight exposure (3.21-5.34 h/d).

Sensitivity analyzes also revealed a positively significant relationship of categorized PM_2.5_ exposure with obesity risk (*P* value for trend <0.001), and the OR increased gradually across the categories, except for abdominal obesity in the highest exposure group (**Supplemental Figure 1**). The findings were stable after adding other potential confounders to the multivariable models (**Table S2**). The association of the one-year mean PM_2.5_ with the risk of obesity and abdominal obesity showed no substantial change of the risk estimates as to the five-year mean PM_2.5_ (**Table S3**). The association assessed by logistics models was also similar to the association assessed by GAMs (**Table S4**). The subgroup analyses suggested that the obesity risk attributed to PM_2.5_ attenuated as the sunshine duration increased, especially in south areas (**Table S5**).

## Discussion

We presented the first study to investigate the effect modification of sunlight on the impact of PM_2.5_ on obesity risk. An inverse J-shaped relationship of PM_2.5_ with obesity and abdominal obesity risk was observed as the sunshine duration increased, with the lowest risk at the middle sunshine duration group (7.18-8.37 h/d) and the highest risk at the bottom quartile (3.21-5.34 h/d).

Most previous studies revealed a positively significant relationship of PM_2.5_ level with obesity (6, 19–24). However, several cross-sectional surveys revealed no association between PM_2.5_ and obesity (25–27), which could be explained by the relatively low level of PM_2.5_ exposure. For instance, in the Framingham Heart Study (27), the annual mean PM_2.5_ level reached 10.6 μg/m^3^, which closely came to the standard from World Health Organization (yearly mean PM_2.5_: 10μg/m^3^). Additionally, several studies reporting no association between PM_2.5_ and obesity mainly focused on subjects living in the area with an abundant sun exposure (25, 26). Our study extended previous observation between PM_2.5_ and obesity to regions with relatively high level of PM_2.5_ exposure. Besides, previous evidence revealed that the risk of abdominal obesity was higher with a high PM_2.5_ concentration in rural areas (19), and short-term exposure has been linked to abdominal obesity (23). Our study further identified a positively significant relationship of long-term effect of PM_2.5_ with abdominal obesity risk.

We observed that the relationship of PM_2.5_ with obesity was influenced by the sunshine duration. Previous studies suggested that sunlight exposure might promote the synthesis of vitamin D and nitric oxide, which in turn could lead to a reduced risk of adiposity accumulation (8, 28). In addition, sunlight exposure may stimulate browning recruitment in white adipose tissue *via* over-expression of cyclooxygenase-2 (COX-2) (29) and facilitated systemic energy expenditure through a myogenic factor 5 (myf-5) independent pathway (30). A cross-sectional study found that the elevated January sunshine duration was associated with an decreased risk of obesity (7). Furthermore, recent studies using latitude (31) and altitude as substitutes for sunlight exposure indicated that decreased latitude (32) or increased altitude (33) (substituting for elevated sunlight exposure) was related to a reduced risk of obesity. However, excess exposure to ultraviolet radiation could overwhelm the cutaneous antioxidant capacity, leading to inflammation and oxidative stress (8, 34). Therefore, sunshine duration exceeding the appropriate range may associate with an elevated risk of obesity, which is consistent with our results.

Experimental studies suggest possible mechanisms for the effect modification of sunlight on the PM_2.5_ in the pathogenesis of obesity. *In vitro* and *in vivo* experiments revealed that both of PM_2.5_ and sunlight exposure induced the reactive oxygen species (ROS) generation and the expression of COX-2 gene (29, 35, 36), while with various pathways. PM_2.5_ could induce generation of the highly reactive hydroxyl radical through catalyzing Fenton’s reaction (37, 38), and could activate COX-2 expression through ROS-nuclear factor kappa B (NF-κB) pathway (39). Meanwhile, sunlight exposure could stimulate ROS production by activating the catalase and promoting nitric oxide synthase synthesis (35), and could activate the expression of COX-2 *via* protein-tyrosine phosphorylation (40). Both excessive ROS production and COX-2 activation led to the progress of systematic inflammation, insulin resistance, and increased oxidative stress (41–43), contributing to an elevated risk of obesity (44, 45).

The study has strengths that deserve mention. The major strength is that the participants were enrolled from multiple centers with relatively high PM_2.5_ levels and the striking latitude gradient. This study has limitations as well. First, our study population was derived from pre-existing cross-sectional study. Causal inferences on effect of PM_2.5_ and sunlight on the risk of obesity or abdominal obesity could not be made because this study did not capture the obesity status prior to exposure. Second, we didn’t assess the effects of gaseous pollutants except for NO_2_. However, NO_2_ was one of the major predictors of health effects and was highly correlated with other gaseous pollutants (46). Third, our assessment of PM_2.5_ and sunlight exposure were ascertained based on the nominal levels but not measured levels, which is common in big data surveys. Fourth, we did not collect the information on the workplace, which leads to the effect evaluation of environmental exposure only based on home address. Fifth, we did not take the effect of potential discrepancy in PM_2.5_ components as a variable into consideration. Furthermore, the possibility of residual confounding could not be excluded.

In conclusion, the present research reveals that the relationship between PM_2.5_ and obesity or abdominal obesity risk varies by sunshine duration, and stronger relationship were observed in short-sunshine-time regions compared to medium- and long-sunshine-time regions. An improved understanding of this interaction effect may offer important insights for lowering obesity risk attributed to environmental factors.

## Data Availability Statement

The data analyzed in this study is subject to the following licenses/restrictions: Due to the nature of this research, participants of this study did not agree for their data to be shared publicly, so supporting data is not available. Requests to access these datasets should be directed to zhanglx@bjmu.edu.cn.

## Ethics Statement

The studies involving human participants were reviewed and approved by the ethics committee at Peking University First Hospital. The patients/participants provided their written informed consent to participate in this study.

## Author Contributions

RC and LZ contributed to the conception and design of the study. RC and CY contributed to the literature review. RC, CY, PL, JW, and LZ contributed to the data collection and data quality control. ZL, WW, YueW, and CL provided the air pollution exposure data. YanW collected the meteorological data. RC, CY, JW, and LZ cleaned, analysed, and visualized the data. RC, CY, JW, and LZ supervised the analysis and generation of results, and directly accessed and verified the data. RC wrote the manuscript. LZ and CY reviewed and edited the manuscript. All authors contributed to data interpretation, and reviewed and approved the final version manuscript. All authors had full access to all the data in the study and had final responsibility for the decision to submit for publication.

## Funding

This study was supported by grants from the National Natural Science Foundation of China (91846101, 82003529, 81771938, 81900665, 82090021), Beijing Nova Programme Interdisciplinary Cooperation Project (Z191100001119008), National Key R&D Program of the Ministry of Science and Technology of China (2019YFC2005000), Chinese Scientific and Technical Innovation Project 2030 (2018AAA0102100), the University of Michigan Health System-Peking University Health Science Center Joint Institute for Translational and Clinical Research (BMU2018JI012, BMU2019JI005, 71017Y2027), CAMS Innovation Fund for Medical Sciences (2019-I2M-5-046), and PKU-Baidu Fund (2019BD017, 2020BD032).

## Conflict of Interest

The authors declare that the research was conducted in the absence of any commercial or financial relationships that could be construed as a potential conflict of interest.

## Publisher’s Note

All claims expressed in this article are solely those of the authors and do not necessarily represent those of their affiliated organizations, or those of the publisher, the editors and the reviewers. Any product that may be evaluated in this article, or claim that may be made by its manufacturer, is not guaranteed or endorsed by the publisher.

## Abbreviations

PM_2.5_, fine particulate matter; WC, waist circumference; WHR, waist-to-hip ratio; OR, odds ratio; CI, confidence interval; BMI, body mass index; SRS, satellite remote sensing; NO_2_, nitrogen dioxide; GAM, general additive model; COX-2, cyclooxygenase-2; myf-5, myogenic factor 5; ROS, reactive oxygen species; NF-κB, nuclear factor kappa B.
