# Supplementary material for: Long-Term Exposure to Ambient PM2.5, Sunlight, and Obesity: A Nationwide Study in China
Source: Front Endocrinol (Lausanne). 2022 Jan 7;12:790294. doi: 10.3389/fendo.2021.790294 (PMC8777285; doi:10.3389/fendo.2021.790294)
Supplement: Supplementary file 1 [file DataSheet_1.docx]

**Supplementary Material**

**Table S1**. Characteristics of mean sunshine duration and PM_2.5_ level by each province

|  | 5-year mean PM_2.5_ (µg/m^3^) | 5-year mean sunshine duration (h/d) |
| --- | --- | --- |
| Beijing | 57.52 (11.78) | 7.92 (0.41) |
| Shanghai | 51.49 (1.33) | 6.32 (0.01) |
| Shandong | 54.44 (1.45) | 7.44 (0.05) |
| Jiangsu | 57.76 (4.79) | 6.67 (0.01) |
| Zhejiang | 53.73 (2.16) | 6.55 (0.04) |
| Guangdong | 40.62 (4.60) | 5.44 (0.22) |
| Hubei | 26.50 (0.60) | 7.18 (0.01) |
| Hunan | 42.67 (0.40) | 7.18 (0.00) |
| Inner Mongolia | 22.12 (1.29) | 9.30 (0.00) |
| Ningxia | 17.71 (2.16) | 9.14 (0.02) |
| Sichuan | 55.76 (3.03) | 3.21 (0.00) |
| Xinjiang | 13.56 (0.15) | 8.78 (0.00) |
| Guangxi | 39.51 (2.62) | 5.11 (0.13) |

Data are presented as mean (SD).

**Table S2.** Estimated effects of 5-year mean PM_2.5_ (10μg/m^3^) with obesity and abdominal obesity risk when controlling for different potential confounders

|  | Obesity | | Abdominal Obesity | | |
| --- | --- | --- | --- | --- | --- |
|  | **OR (95%CI)** | ***P*-value** | **OR (95%CI)** | | ***P*-value** |
| Crude OR | 1.07 (1.05,1.10) | <0.001 | 1.03 (1.02,1.04) | <0.001 | |
| Model 1 | 1.06 (1.04,1.09) | <0.001 | 1.01 (0.99,1.03) | 0.332 | |
| Model 2 | 1.03 (1.01,1.05) | 0.003 | 1.01 (0.99,1.03) | 0.199 | |
| Model 3 | 1.12 (1.09,1.14) | <0.001 | 1.10 (1.07,1.13) | <0.001 | |
| Model 4 | 1.12 (1.10,1.15) | <0.001 | 1.10 (1.08,1.13) | <0.001 | |
| Model 5 | 1.12 (1.10,1.14) | <0.001 | 1.10 (1.08,1.13) | <0.001 | |
| Model 6 | 1.12 (1.09,1.14) | <0.001 | 1.10 (1.08,1.13) | <0.001 | |

Model 1: Age, sex, and NO_2_.

Model 2: Age, sex, educational background, smoker, intake of alcohol, household income, rural, and NO_2_.

Model 3: Age, sex, educational background, smoker, intake of alcohol, household income, rural, NO_2_, and sunlight hours.

Model 4: Age, sex, educational background, smoker, intake of alcohol, household income, rural, exercise duration, NO_2_, and sunlight hours.

Model 5: Age, sex, educational background, smoker, intake of alcohol, household income, rural, fruit and vegetable diet, daily protein intake, NO_2_, and sunlight hours.

Model 6: Age, sex, educational background, smoker, intake of alcohol, household income, rural, exercise duration, daily protein intake, fruit and vegetable diet, NO_2_, and sunlight hours.

**Table S3**. Estimated effects of 1-year mean PM_2.5_ (10μg/m^3^) with obesity and abdominal obesity risk

|  | Obesity | | | Abdominal Obesity | | |
| --- | --- | --- | --- | --- | --- | --- |
|  | **OR (95%CI)** | | ***P*-value** | **OR (95%CI)** | | ***P*-value** |
| Overall | |  |  |  |  | |
| Crude OR | 1.07 (1.05,1.09) | | <0.001 | 1.02 (1.01,1.04) | <0.001 | |
| Model 1 | 1.06 (1.04,1.09) | | <0.001 | 1.00 (0.98,1.02) | 0.803 | |
| Model 2 | 1.02 (1.00,1.04) | | 0.001 | 1.01 (1.00,1.03) | 0.025 | |
| Model 3 | 1.12 (1.10,1.14) | | <0.001 | 1.10 (1.08,1.13) | <0.001 | |

Model 1: Age, sex, and NO_2_.

Model 2: Age, sex, educational background, smoker, intake of alcohol, household income, rural, and NO_2_.

Model 3: Age, sex, educational background, smoker, intake of alcohol, household income, rural, NO_2_, and sunlight hours.

**Table S4.** Estimated effects of 5-year mean PM_2.5_ (10μg/m^3^) on the risk of obesity and abdominal obesity in China by logistics models

|  | Obesity | | | | Abdominal obesity | | |
| --- | --- | --- | --- | --- | --- | --- | --- |
|  | **OR (95% CI)** | | | ***P*-value** | **OR (95% CI)** | | ***P*-value** |
| Overall | | |  |  |  |  | |
| Crude OR | 1.08 (1.04,1.12) | | | <0.001 | 1.05 (1.03,1.07) | <0.001 | |
| Model 1 | 1.07 (1.05,1.09) | | | <0.001 | 1.04 (1.02,1.05) | <0.001 | |
| Model 2 | 1.03 (1.01,1.05) | | | 0.009 | 1.02 (1.00,1.04) | 0.177 | |
| Model 3 | 1.13 (1.11,1.15) | | | <0.001 | 1.11 (1.09,1.14) | <0.001 | |
| Subgroup | |  | |  |  |  | |
| Sunshine duration, h/d | |  | |  |  |  | |
| Q1(3.21-5.34) | 1.67 (1.38,2.03) | | | <0.001 | 1.74 (1.41,2.14) | <0.001 | |
| Q2(5.34-7.18) | 1.24 (1.14,1.35) | | | <0.001 | 1.36 (1.23,1.50) | <0.001 | |
| Q3(7.18-8.37) | 1.03 (0.97,1.08) | | | 0.331 | 0.99 (0.93,1.05) | 0.704 | |
| Q4(8.37-9.30) | 1.05 (1.01,1.09) | | | 0.008 | 1.07 (1.02,1.11) | 0.002 | |

Model 1: Age, sex, and NO_2_.

Model 2: Age, sex, educational background, smoker, intake of alcohol, household income, rural, and NO_2_.

Model 3: Age, sex, educational background, smoker, intake of alcohol, household income, rural, NO_2_, and sunlight hours.

**Table S5.** Estimated effects of 5-year mean PM_2.5_(10μg/m^3^) with obesity and abdominal obesity risk, by south and longitude subgroups

| Sunshine Duration | Obesity | | | Abdominal obesity | | |
| --- | --- | --- | --- | --- | --- | --- |
|  | **OR (95% CI)** | ***P*-value** | **Interaction *P* Value** | **OR (95% CI)** | ***P*-value** | **Interaction *P* Value** |
| South |  |  | 0.034 |  |  | 0.459 |
| Q1(3.21-5.29) | 1.71 (1.34,2.17) | <0.001 |  | 1.78 (1.34,2.35) | <0.001 |  |
| Q2(5.29-5.94) | 1.61 (1.32,1.97) | <0.001 |  | 1.41 (1.15,1.74) | 0.001 |  |
| Q3(5.94-6.55) | 1.82 (0.90,3.69) | 0.098 |  | 1.37 (0.70,2.66) | 0.359 |  |
| Q4(6.55-7.18) | 1.37 (1.24,1.50) | <0.001 |  | 1.43 (1.27,1.61) | <0.001 |  |
| North |  |  | 0.857 |  |  | 0.442 |
| Q1(7.22-7.59) | 1.07 (0.91,1.25) | 0.410 |  | 1.11 (0.94,1.31) | 0.211 |  |
| Q2(7.59-8.37) | 0.98 (0.92, 1.04) | 0.452 |  | 0.98 (0.92,1.04) | 0.490 |  |
| Q3(8.37-9.12) | 1.02 (0.97, 1.07) | 0.378 |  | 0.99 (0.95,1.05) | 0.825 |  |
| Q4(9.12-9.30) | 1.20 (0.74, 1.93) | 0.467 |  | 1.02 (0.61, 1.74) | 0.913 |  |
| Longitude |  |  |  |  |  |  |
| UTC+7 |  |  | <0.001 |  |  | <0.001 |
| Q1(3.21-5.01) | 1.71 (1.34, 2.17) | <0.001 |  | 1.94 (1.46, 2.58) | <0.001 |  |
| Q2(5.01-5.34) | 1.22 (1.01, 1.49) | 0.041 |  | 1.29 (1.10, 1.52) | 0.002 |  |
| Q3(5.34-7.18) | 1.02 (0.96, 1.08) | 0.245 |  | 1.20 (0.95, 1.53) | 0.132 |  |
| Q4(7.18-9.16) | 0.99 (0.95, 1.04) | 0.727 |  | 1.01 (1.00, 1.02) | 0.038 |  |
| UTC+8 |  |  | <0.001 |  |  | <0.001 |
| Q1(6.32-6.67) | 1.21 (1.03, 1.42) | 0.018 |  | 1.19 (1.01, 1.41) | 0.039 |  |
| Q2(6.67-7.51) | 1.24 (1.00, 1.45) | 0.007 |  | 1.11 (0.94, 1.31) | 0.211 |  |
| Q3(7.51-8.37) | 1.04 (0.98, 1.10) | 0.190 |  | 0.98 (0.92, 1.04) | 0.490 |  |
| Q4(8.37-9.30) | 1.01 (0.97, 1.05) | 0.697 |  | 1.03 (0.98, 1.07) | 0.292 |  |

Model: Age, sex, educational background, smoker, intake of alcohol, household income, rural, NO_2_, and sunlight hours.

Note: Longitude subgroups range from 97.5ºE to 112.5ºE (UTC+7) and 112.5ºE to 127.5ºE (UTC+8), respectively.

South or north region represents latitude subgroups.

**Figure legends**

**Supplemental Figure 1**. Adjusted odds ratios of obesity and abdominal obesity related to categorized PM_2.5_ exposure. (A)obesity; (B) abdominal obesity.

Group 1 is the reference group. The odds ratio (Figure 1A) for group 2 is 1.33 (95%CI, 1.19-1.48), group 3 is 1.62 (95%CI, 1.43-1.84), and group 4 is 1.65 (95%CI, 1.50-1.82). The odds ratio (Figure 1B) for group 2 is 1.29 (95%CI, 1.14-1.45), group 3 is 1.71 (95%CI, 1.49-1.96), and group 4 is 1.55 (95%CI, 1.40-1.73).

Model: Age, sex, educational background, smoker, intake of alcohol, household income, rural, NO_2_, and sunlight hours.
