# Supplementary figures and images for: Long-Term Exposure to Ambient PM2.5, Sunlight, and Obesity: A Nationwide Study in China
Source: Front Endocrinol (Lausanne). 2022 Jan 7;12:790294. doi: 10.3389/fendo.2021.790294 (PMC8777285; doi:10.3389/fendo.2021.790294)

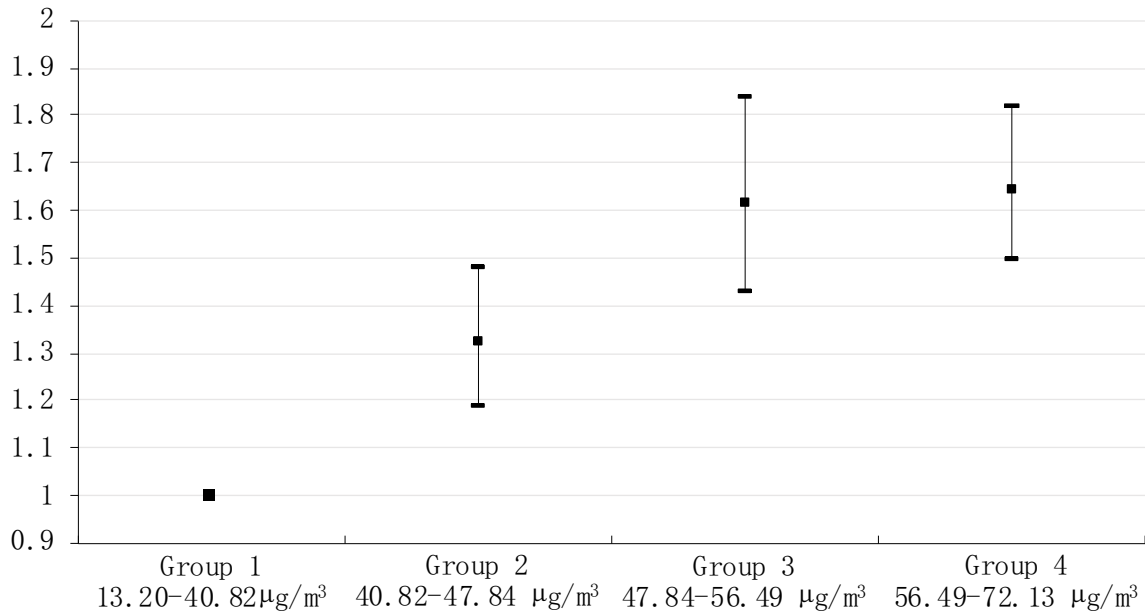

Supplement: Supplementary file 2 [file Image_1.pdf]

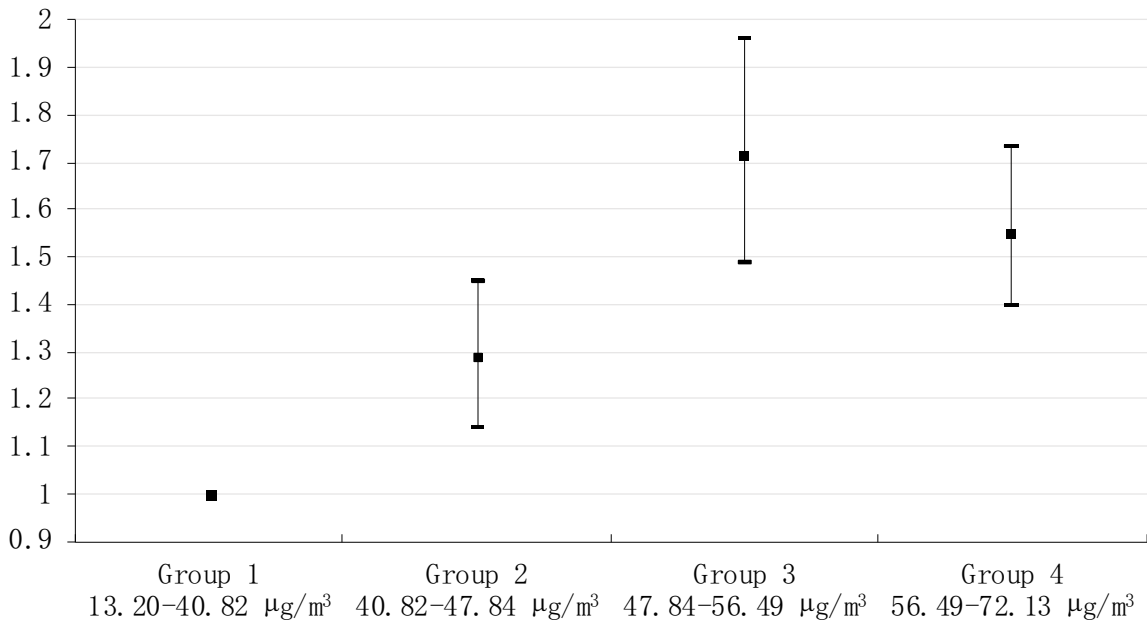

Supplement: Supplementary file 3 [file Image_2.pdf]
